# Acupoint Injection of Onabotulinumtoxin A for Migraines

**DOI:** 10.3390/toxins7114442

**Published:** 2015-10-30

**Authors:** Min Hou, Jun-Fan Xie, Xiang-Pan Kong, Yi Zhang, Yu-Feng Shao, Can Wang, Wen-Ting Ren, Guang-Fu Cui, Le Xin, Yi-Ping Hou

**Affiliations:** 1 Department of Neuroscience, Anatomy, Histology and Embryology, Key Laboratory of Preclinical Study for New Drugs of Gansu Province, School of Basic Medical Sciences, Lanzhou University, 199 Donggang Xi Road, Lanzhou 730000, China; E-Mails: sunny_smile_824@163.com (M.H.); xiejfan@163.com (J.-F.X.); kongxp530@163.com (X.-P.K.); shaoyf@lzu.edu.cn (Y.-F.S.); wangc2012@lzu.cn (C.W.); renwt13@163.com (W.-T.R.); cgf0204@163.com (G.-F.C.); xinle9058@163.com (L.X.); 2 Department of Anatomy, Gansu University of Traditional Chinese Medicine, Lanzhou 730000, China; 3 Department of Human Anatomy, School of Medicine, Hunan Normal University, 371 Tongzipo Road, Changsha 410013, China; 4 Department of Neurology and Pain Treatment, Gansu Province People Hospital, Lanzhou 730000, China; E-Mail: zhangyi9310@21cn.com

**Keywords:** botulinum toxin type A, migraine, fixed-sites injection, acupoint-sites injection, randomized and placebo-controlled trial

## Abstract

Onabotulinumtoxin A (BoNTA) has been reported to be effective in the therapy for migraines. Acupuncture has been used worldwide for the treatment of migraine attacks. Injection of a small amount of drug at acupuncture points is an innovation as compared to traditional acupuncture. The purpose of this study was to evaluate and compare the effectiveness of fixed (muscle)-site and acupoint-site injections of BoNTA for migraine therapy in a randomized, double-blinded, placebo-controlled clinical trial extending over four months. Subjects with both episodic and chronic migraines respectively received a placebo (*n* = 19) or BoNTA (2.5 U each site, 25 U per subject) injection at fixed-sites (*n* = 41) including occipitofrontalis, corrugator supercilii, temporalis and trapeziue, or at acupoint-sites (*n* = 42) including Yintang (EX-HN3), Taiyang (EX-HN5), Baihui (GV20), Shuaigu (GB8), Fengchi (GB20) and Tianzhu (BL10). The variations between baseline and BoNTA post-injection for four months were calculated monthly as outcome measures. BoNTA injections at fixed-sites and acupoint-sites significantly reduced the migraine attack frequency, intensity, duration and associated symptoms for four months compared with placebo (*p* < 0.01). The efficacy of BoNTA for migraines in the acupoint-site group (93% improvement) was more significant than that in the fixed-site group (85% improvement) (*p* < 0.01). BoNTA administration for migraines is effective, and at acupoint-sites shows more efficacy than at fixed-sites. Further blinded studies are necessary to establish the efficacy of a low dose toxin (25 U) introduced with this methodology in chronic and episodic migraines.

## 1. Introduction

Migraines are a common primary headache disorder, which affects 15% of the world’s population [1] and is typically characterized as a neurovascular disorder of recurring, throbbing headaches, and often associated with aura, nausea, vomiting, photophobia, phonophobia, fatigue and enhanced irritability [2,3]. Both pericranial muscle tenderness (muscle allodynia) and cutaneous allodynia (scalp allodynia) have also been described during migraine attacks [4,5]. Nociceptive inputs of myofascial origin have been postulated to play a role in migraine pathogenesis [6].

Migraines are a complex, neurovascular disorder in which genetic and environmental factors interact [7]. One theory, based on preclinical observations, is that activation of trigeminal sensory fibers leads to a painful neurogenic inflammation within the meningeal vasculature mediated by neuropeptide release from trigeminal sensory fibers and characterized by plasma protein extravasation, vasodilation, and mast cell degranulation [8]. Previous experiments have demonstrated that unmyelihated nociceptive trigeminal fibers provide the major source of sensory innervation for cranial blood vessels (trigeminovascular system) [9] and contain neuropeptides such as calcitonin gene-related peptide (CGRP) and substance P (SP) [9]. These neurons convey nociceptive impulses to the brain and at the same time may co-release CGRP and SP from the peripheral endings, thus evoking a variety of effects collectively known as neurogenic inflammation [10]. The hypothesis that the activation, for unknown reasons, of peptide-containing trigeminal neurons leads to the generation of pain and simultaneous inflammatory effects in cranial blood vessels has the advantage of offering a unifying explanation for the crucial symptoms of migraine, such as pain and vasodilation [10].

Onabotulinumtoxin A (BoNTA) is a focally administered neurotoxin, which inhibits the release of acetylcholine at the neuromuscular junction and is used therapeutically in disorders characterized by muscle hyperactivity [11]. It has also been reported to be effective in the therapy for migraine [12,13,14] or chronic migraine [15,16,17,18]. Until now, most researchers have based reports of improvements in the symptoms of migraineurs with BoNTA injections on studies in which fixed-sites in the forehead, temple and neck muscles are injected for muscle-relaxing [12,14,19,20,21]. New evidence has recently demonstrated that acupoint-site injection of BoNTA is useful in alleviating or eliminating symptoms of migraine [22,23,24]. In traditional Chinese medicine, acupuncture is considered one of the most effective treatments for migraines [25,26,27]. Acupoints are believed to correspond to energy channels that circulate through the body and connect the organs and the viscera [28]. Acupoint injection as a therapeutic technique is well documented and indicated to have quicker and more powerful clinical effects than muscle and subcutaneous injection [29] and has been used to treat some diseases including headaches [30], myofascial pain [31], temporomandibular disorders [32], rheumatoid arthritis [33] and hip dysplasia [34], although the mechanism of acupoint injection is unclear.

The purpose of the present study in a randomized, double-blinded, placebo-controlled clinical trial was to investigate the therapeutic effect of BoNTA on migraines, and evaluate the efficacy between fixed-sited and acupoint-sited injection of BoNTA.

## 2. Results and Discussion

### 2.1. Subjects’ Characteristics

In this study, 102 Chinese subjects were enrolled (79.4% female), and 35.3% of them were sufferers of chronic migraines. The mean age of enrolled subjects was 40.7 ± 9.0 years. The baseline characteristics including age, sex, weight and the duration of migraine in three groups were not significantly different (Table 1). There were no statistically significant differences in migraine severity, distribution (unilateral *versus* bilateral), type of pain, or effect of physical activity between placebo and BoNTA injection in fixed-sites and acupoint-sites groups before treatment.

**Table 1:** Subjects’ characteristics.

Group		Case (n)	Gender M/F (n)	Age (y)	Weight (kg)	Stature (cm)	Duration of Migraine (y)
Placebo		19	4/15	41.7 ± 8.8	58.7 ± 5.8	159.1 ± 7.4	6.8 ± 2.6
BoNTA	Fixed-sites	41	8/33	39.8 ± 9.2	57.4 ± 7.1	158.3 ± 7.5	5.0 ± 2.9
	Acupoint-sites	42	9/33	41.0 ± 9.1	59.7 ± 6.6	160.9 ± 7.0	6.1 ± 3.6
Total		102	21/81	40.7 ± 9.0	58.6 ± 6.7	159.5 ± 7.3	6.0 ± 3.6

### 2.2. Effect of BoNTA Injection on the Attack Frequency, Intensity, Duration and Associated Symptoms of Migraines

BoNTA injection in both of the fixed-sites and acupoint-sites groups induced a significant decrease of the attack frequency of migraine for four months compared with placebo (*p* < 0.01; Table 2). However, the reduction of attack frequency in acupoint-sites group was greater from month 1 to 4 (*p* < 0.01; Table 2) compared with fixed-sites group.

**Table 2:** Attack frequency of migraines (numbers/month).

Group		Baseline	Post-Injection (Month)			
			1	2	3	4
Placebo		7.5 ± 2.0	7.4 ± 3.2	7.1 ± 3.3	7.2 ± 3.0	7.4 ± 2.8
BoNTA	Fixed-sites	7.2 ± 1.7	4.0 ± 1.4 *	3.8 ± 1.6 *	3.6 ± 1.4 *	3.7 ± 1.3 *
	Acupoint-sites	7.6 ± 2.1	2.1 ± 1.0 *^,†^	1.8 ± 0.9 *^,†^	1.7 ± 0.8 *^,†^	1.9 ± 1.0 *^,†^

* *p* < 0.01, compared with placebo; ^†^ *p* < 0.01, compared with fixed-sites.

As shown in Table 3, the reduction in migraine intensity was distinct from month 1 to 4 in both of the BoNTA treated groups compared to placebo, respectively (*p* < 0.01; Table 3). In addition, the reduction of migraine intensity in acupoint-sites group was statistically different from fixed-sites group for four months (*p* < 0.01; Table 3).

**Table 3:** Intensity of migraines (visual analogue scale (VAS) 0–10)).

Group		Baseline	Post-Injection (Month)			
			1	2	3	4
Placebo		6.9 ± 1.9	6.6 ± 2.1	6.1 ± 2.3	6.3 ± 2.2	6.4 ± 2.1
BoNTA	Fixed-sites	6.8 ± 1.6	4.2 ± 1.4 *	4.0 ± 1.3 *	3.8 ± 1.5 *	4.0 ± 1.4 *
	Acupoint-sites	7.0 ± 1.5	2.1 ± 1.0 *^†^	1.9 ± 0.9 *^,†^	1.8 ± 0.8 *^,†^	2.0 ± 0.9 *^,†^

* *p* < 0.01, compared with placebo; ^†^ *p* < 0.01, compared with fixed-sites.

The mean duration of each attack after BoNTA treatment in both groups was significantly reduced compared to that in placebo (*p* < 0.01; Table 4). In comparison between fixed-sites and acupoint-sites groups, the decrease of each attack duration in acupoint-sites group was greater for four months (*p* < 0.01; Table 4).

**Table 4:** Duration of migraine (hours/once).

Group		Baseline	Post-Injection (Month)			
			1	2	3	4
Placebo		7.4 ± 2.1	7.3 ± 2.5	7.1 ± 2.6	7.2 ± 2.4	7.2 ± 2.2
BoNTA	Fixed-sites	7.0 ± 2.4	3.8 ± 1.2 *	3.1 ± 1.3 *	3.0 ± 1.2 *	3.5 ± 1.4 *
	Acupoint-sites	7.9 ± 2.6	2.0 ± 0.9 *^,†^	1.5 ± 0.7 *^,†^	1.4 ± 0.7 *^,†^	1.6 ± 0.7 *^,†^

* *p* < 0.01, compared with placebo; ^†^ *p* < 0.01, compared with fixed-sites.

Most participants before BoNTA injection experienced at least one of the four symptoms typically associated with a migraine attack (*i.e.*, vomiting, nausea, photophobia, phonophobia). The decrease of migraine-associated symptoms in both groups after BoNTA treatment was statistically significant for four months compared with placebo (*p* < 0.01; Table 5). In comparison between different sites of injection, the reduction of migraine-associated symptoms in acupoint-sites group was statistically different from fixed-sites group from month 1 to 4 (*p* < 0.01; Table 5).

**Table 5:** Associated symptoms of migraine (scores).

Group		Baseline	Post-Injection (Month)			
			1	2	3	4
Placebo		0.72 ± 0.20	0.71 ± 0.22	0.68 ± 0.21	0.69 ± 0.23	0.68 ± 0.21
BoNTA	Fixed-sites	0.74 ± 0.21	0.36 ± 0.14 *	0.33 ± 0.15 *	0.31 ± 0.14 *	0.34 ± 0.15 *
	Acupoint-sites	0.69 ± 0.23	0.18 ± 0.09 *^,†^	0.16 ± 0.09 *^,†^	0.15 ± 0.08 *^,†^	0.18 ± 0.07 *^,†^

* *p* < 0.01, compared with placebo; ^†^ *p* < 0.01, compared with fixed-sites.

### 2.3. Efficiency of Fixed-Sites and Acupiont-Sites Injection of BoNTA

Following BoNTA injection in fixed-sites group, 35 (85%) of 41 subjects noted improvement in migraine for four months, in which nine (22%) were attack-free, 14 (34%) were markedly effective, 12 (29%) were effective and six (15%) were invalid. Whereas, in acupoint-sites group of BoNTA injection, 39 (93%) of 42 patients noted improvement in migraine for four months, in which 14 (33%) were attack-free, 20 (48%) were markedly effective, 5 (12%) were effective and three (7%) were invalid. Data of this study demonstrated that BoNTA injection at acupoint-sites for migraine treatment was more efficient than at fixed-sites (*p* < 0.01).

### 2.4. Side Effects

Three cases (7%) of 41 subjects injected with BoNTA at fixed-sites appeared transient unilateral upper eyelid ptosis that lasted for three or five days. One patient in fixed-sites group after injection noted an acute pain at injection sites that disappeared after one night. These three subjects were among the cases of improvement in fixed-sites group. One patient in acupoint-sites group after injection felt an ethereal pain in local skin that lasted for four days, and BoNTA treatment was invalid for him.

### 2.5. Discussion

The present study demonstrated that injection of BoNTA, wherever into fixed-sites or acupoint-sites could significantly reduce migraine frequency, density, duration and associated symptoms. The beneficial effects of BoNTA were observed mainly from one to four months post-injection, in line with other preventive migraine treatment [8,35]. The improvement on treatments produced by BoNTA suggests that the effect of BoNTA on migraine could be beyond four months.

The 25-U dose of BoNTA for migraine treatment in this study was proved as an efficacious dosage. Although the efficacy of low dose found in the pilot study in China agrees with some previous studies [12,36], it is at odds with the current literature. More recent studies of the Phase III Research Evaluating Migraine Prophylaxis Therapy (PREEMPT) have demonstrated and recommended 155–195 U dose of BoNTA for migraine therapy [37,38]. Considering the inadequacy of this single study, PREEMPT paradigm for injection dose of BoNTA will be used in future protocol of migraine treatment.

The mechanisms involved in BoNTA for migraine therapy are considered to inhibit sensory neurotransmitter releases from the trigeminal neurons that are associated with a concentration-dependent cleavage of synaptosome-associated protein SNAP-25 [39]. A recent study in our laboratory has demonstrated that BoNTA suppresses nitroglycerin-induced migraine-like symptoms and CGRP- and SP-like immunereactivity in the jugular plasma and medulla oblongata in rats, and suggests that the sensory-specific effect of BoNTA might occur through inhibition of sensory neuropeptide vesicle release via a SNAP-mediated mechanism, similar to the blockade of acetylcholine release in motor neurons [24]. This mechanism has been thought to underlie the frequently reported reduction of pain with BoNTA in the treatment of migraine. In addition, Burstein and his colleagues [40] have showed that BoNTA selectively inhibits meningeal nociceptors and prevents or reverses *C*-fibremediated mechanical hypersensitivity, possibly through decreased trafficking of mechanosensitive ion channels to the cell surface. Notably, BoNTA inhibited the mechanical sensitivity of the suture branches of intracranial meningeal nociceptors when administered extracranially [17].

More importantly, our study aims at the comparison of efficacy between fixed-sites and acupoint-sites injection of the same dose of BoNTA for migraine treatment. Our results showed that BoNTA-induced efficient improvement for reducing migraine frequency, density, duration and associated symptoms in acupoint-sites group was 93% and more statistically significant than 85% in fixed-sites groups. This evidence suggests that acupoint-sites injection of BoNTA is efficacious for migraine treatment, and better than fixed-sites injection. Acupuncture is effective and safe in relieving headache, preventing relapse and reducing migraine associated symptoms and has been used worldwide for the treatment of migraine attacks [27,41]. Vernejoul and his colleagues have studied the migration of radioactive tracers injected at acupoint-sites using a scintillation camera coupled to a computer system with image analysis capability [42]. They found the migration of radioactive tracer from one acupoint to another, along pathways superimposable with the traditional Chinese medicine, taking into account the migration speed and few patterns, does not indicate an intravascular lymphatic origin. The evidence of most subjects who reported a more powerful stimulation response with acupoint injection may indicate that the point injection provides a more powerful clinical outcome, with stronger sensations be translated into a stronger clinical response, as outlined by Wang *et al.* [43] and Luo and Chen [44].

Several patients in this study injected with BoNTA had transient unilateral upper eyelid ptosis and injection site pain and recovered quickly without any treatment, confirming the tolerability of BoNTA as reported in the other studies [45]. Other agents used in the prophylaxis of migraines cause side effects such as fatigue, dizziness, reduced concentration, loss of appetite, weight gain, hair loss, and changes in libido [46]. However, BoNTA treatment in this clinical trial has not been reported to cause such side effects.

The majority of controlled studies with BoNTA report failure of this form of treatment in episodic migraine [20,37,47,48]. Controlled studies using our methodology and dose are necessary to see if our positive results can be independently replicated.

## 3. Experimental Section

### 3.1. Study Design

This was a randomized, double-blind, placebo-controlled study of BoNTA compared with placebo as analgesic agent in subjects with migraine, and performed at the management of patients with migraine admitted to Department of Neurology and Pain Treatment, Gansu Province People Hospital, China. According to a predetermined computer-made randomization list, the eligible patients were assigned to placebo (*n* = 19), fixed (muscle)-sites injection of BoNTA (*n* = 41) or to acupoint-sites injection of BoNTA (*n* = 42). The patients had an equal probability of being assigned to either treatment groups. Each patient was asked, before enrollment, to give an informed consent to participate in the study. For one month prior to and four months following treatment, subjects recorded the following headache parameters that contained 56 item in diaries provided by investigators: occurrence of migraine and nonmigraine headaches, the start and stop time of migraine, migraine severity, the presence of migraine aura, migraine associated symptoms, and acute migraine medications or treatment used.

The follow-up protocol was identical to that used during the blinded study. This study was conducted in compliance with institutional review broad regulations, informed consent regulations, the Declaration of Helsinki, and the International Headache Society (IHS) guidelines 2004 [49].

### 3.2. Participant Characteristics

Subjects with both episodic and chronic migraine, both genders (21 men and 81 women), aged 18 to 57 years, who had a history of IHS-defined migraines with or without aura for one to 16 years, were eligible to participate. Subjects experienced headache ≥15 days per month for less than or equal to months were diagnosed as chronic migraine [3]. Women of childbearing potential were required to be taking approved birth control measures and to have a negative urine pregnancy test prior to administration of study medications. All eligible subjects were required to be able to understand the study instructions, complete all questionnaires, and maintain a daily diary record of migraine as well as be willing to give informed consent.

Subjects were excluded from the study if they had any medical or neurological conditions that might put them at risk with BoNTA exposure, such as amyotrophic lateral sclerosis, myasthenia gravis, Eaton-Lambert Syndrome. Subjects that had any disease that might interfere with neuromuscular function, had abnormal pathology contributing to migraine, had uncontrolled systemic disease, and had concurrent infection at proposed injection sites, or were pregnant or breast-feeding were considered ineligible for this study. Subjects were also excluded if they were currently using aminoglycoside antibiotics, curare-like agents, or other agents that might interfere with neuromuscular function; had undergone injection of anesthetics or steroids, within one month immediately prior to enrollment, into the muscles to be injected in the study; or had previously received BoNT treatment with any serotype. Subjects were also excluded if they were currently participating in another drug or device study or had done so within the 30 days before the baseline period, had suspected hypersensitivity to BoNTA or any of the ingredients in the proprietary formulation, or had known or suspected drug or alcohol abuse.

### 3.3. Treatment

**Figure 1:**
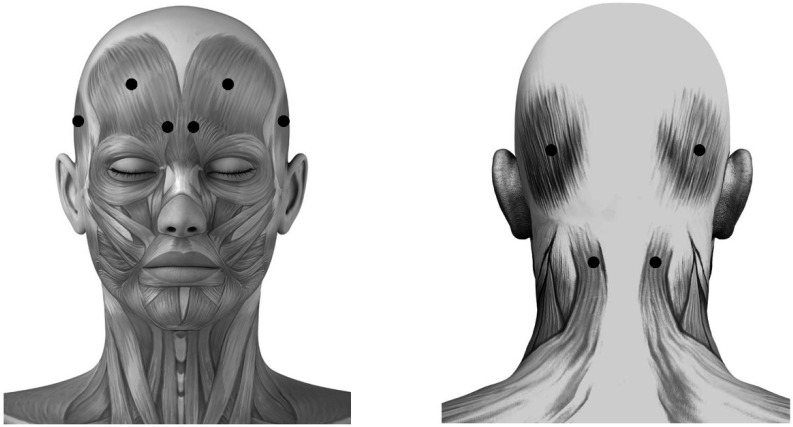
Schematic sites of fixed-sites injection. These sites (●) are respectively located at frontal and occipital belly of occipitofrontalis, corrugator supercilii, temporalis and superior part of trapeziue muscle.

Subjects were randomized 2:2:1 to receive fixed-sites, or acupoint-sites of BoNTA or placebo injections. In order to maintain the double-blind protocol, a research coordinator who was not directly involved with the subjects managed the randomization and preparation of injections. The application sites of placebo and fixed-sites groups were respectively at frontal and occipital belly of occipitofrontalis, corrugator supercilii, temporalis and superior part of trapeziue (Figure 1), which have been mostly selected to treat migraine with BoNTA in previous studies [12,14,19,45,50].

**Figure 2:**
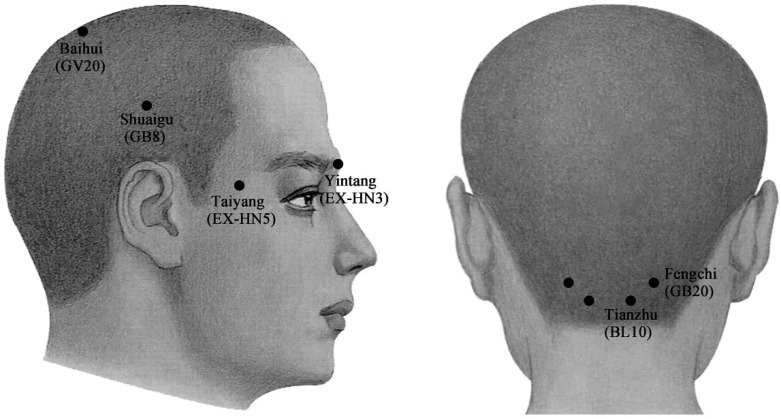
Schematic sites of acupoint-sites injection. These sites (●) are Yintang (EX-HN3), at the midpoint of the line connecting the two medial ends of eyebrows; Taiyang (EX-HN5), at the point of intersection of the continuations of the eyebrow and the lower eyelid in the lateral direction, on the lateral border of the orbit; Baihui (GV20), at the middle of the vertex, on the line connecting the apexes of the two ears; Shuaigu (GB8), directly above the ear apex, 1.5 inches above the hairline; Fengchi (GB20), at the posterior lateral aspect of the neck, in the fossa between the superior margins of the trapezius and sternocleidomastoid muscles; and Tianzhu (BL10), 1.3 inches lateral to the point 0.5 inches directly above the midpoint of the posterior hairline, in the depression lateral to the border of the trapezius muscle.

The injection sites in acupoint groups were respectively at Yintang (EX-HN3), Taiyang (EX-HN5), Baihui (GV20), Shuaigu (GB8), Fengchi (GB20) and Tianzhu (BL10), based on predetermined and well-known Chinese acupoints for the treatment of migraines (Figure 2) [51].

When subjects in three groups finished the prescribed diary of baseline, they were administered 0.1 mL of 0.9% sterile saline as placebo, or 0.1 mL of saline containing BoNTA 2.5 U for a total dose of 25 U per subject through a 1-inch, 30-gauge needle into each site. All of the injections were respectively performed by three of the authors (Min Hou, Yu-Feng Shao and Yi Zhang) to assure consistency.

HENGLI^®^ (Lanzhou Institute of Biological Products Co. Ltd., Lanzhou, Gansu, China) was used in this study, though the units are not truly comparable between toxins an approximation of 1 to 1 U BOTOX (Allergan, Irvine, CA, USA) to 3–4 U Dysport (Ipsen, Slough, UK) has been used in the literature [52]. Each vial of HENLI^®^ contained 100 U of Clostridium botulinum toxin type A, and was prepared freshly with 0.9% sterile saline for a dilution of 2.5 U/0.1 mL. Each vial of placebo contained 0.9% sterile saline. The reconstituted BoNTA or placebo was used within 4 h of injection; it was stored in a refrigerator (4 °C) if not used immediately.

### 3.4. Outcome Measure

The analysis of the diary data was conducted by investigators who did not know the exact group of each subject. Diaries were collected monthly by mail. Evaluation of the different parameters was carried out monthly such as: (I) Frequency was recorded as number of migraine attack per month; (II) Intensity of migraine was measured on a visual analogue scale (VAS) ranging from 0 to 10:0, no pain; 10, unbearable pain; 1–3, mild; 4–6 moderate; 7–9, severe pain; (III) Duration of each attack; (IV) Migraine associated symptoms scale ranging from 0 to 3:0, lack; 3, three to four; 2, two; 1, one of symptoms including vomiting, nausea, phonophobia and photophobia. Their variations in respect to the baseline (one month prior to treatment) were calculated every month as outcome measures.

### 3.5. Statistical Analyses

The data were analyzed by one of the authors (Jun-Fan Xie) who did not participate in group division and treatment. The values were presented as means ± SD. The data of subjects’ characteristics were analyzed by one-way ANOVA. Multivariate ANOVA was used for comparing quantitative data of migraine attack frequency, intensity, duration and associated symptoms in baseline and post-injection among groups, when indicated by significant ANOVA, *post hoc* comparison among means were conducted with LSD. For binomial qualitative data, comparisons between BoNTA treatment groups were done with chi-square tests. Differences between means were considered significant at *p* < 0.05.

## 4. Conclusions

It can be concluded that acupoint-site and fixed-site injection of BoNTA are able to significantly reduce migraine attack frequency, intensity, duration and associated symptoms. Acupoint-site administration of BoNTA is proved to be more efficient for migraines than fixed-site application, and thus is a potential method in clinical practices in treating patients who experienced six or more attacks per month, hemiplegic and basilar type migraines, and migraines with prolonged auras as well.

## References

[1] Jensen R., Stovner L.J. (2008). Epidemiology and comorbidity of headache. Lancet Neurol..

[2] Jensen K. (1993). Extracranial blood flow, pain and tenderness in migraine. Clinical and experimental studies. Acta Neurol. Scand. Suppl..

[3] Olesen J., Bendtsen L., Dodick D., Ducros A., Evers S., First M., Goadsby P.J., Hershey A., Katsarava Z., Levin M. (2013). The international classification of headache disorders, 3rd edition. Cephalalgia.

[4] Burstein R., Yarnitsky D., Goor-Aryeh I., Ransil B.J., Bajwa Z.H. (2000). An association between migraine and cutaneous allodynia. Ann. Neurol..

[5] Mathew N.T., Kailasam J., Seifert T. (2004). Clinical recognition of allodynia in migraine. Neurology.

[6] Olesen J. (1991). Clinical and pathophysiological observations in migraine and tension-type headache explained by integration of vascular, supraspinal and myofascial inputs. Pain.

[7] Farinelli I., De Filippis S., Coloprisco G., Missori S., Martelletti P. (2009). Future drugs for migraine. Intern. Emerg. Med..

[8] Williamson D.J., Hargreaves R.J. (2001). Neurogenic inflammation in the context of migraine. Microsc. Res. Tech..

[9] May A., Goadsby P.J. (2001). Substance p receptor antagonists in the therapy of migraine. Expert Opin. Investig. Drugs.

[10] Fanciullacci M., Alessandri M., Figini M., Geppetti P., Michelacci S. (1995). Increase in plasma calcitonin gene-related peptide from the extracerebral circulation during nitroglycerin-induced cluster headache attack. Pain.

[11] Dolly O. (2003). Synaptic transmission: Inhibition of neurotransmitter release by botulinum toxins. Headache.

[12] Silberstein S., Mathew N., Saper J., Jenkins S. (2000). Botulinum toxin type A as a migraine preventive treatment. For the BOTOX migraine clinical research group. Headache.

[13] Behmand R.A., Tucker T., Guyuron B. (2003). Single-site botulinum toxin type A injection for elimination of migraine trigger points. Headache.

[14] Diener H.C., Dodick D.W., Goadsby P.J., Lipton R.B., Olesen J., Silberstein S.D. (2011). Chronic migraine—Classification, characteristics and treatment. Nat. Rev. Neurol..

[15] Farinelli I., Coloprisco G., De Filippis S., Martelletti P. (2006). Long-term benefits of botulinum toxin type A (BOTOX) in chronic daily headache: A five-year long experience. J. Headache Pain.

[16] Ashkenazi A. (2010). Botulinum toxin type A for chronic migraine. Curr. Neurol. Neurosci. Rep..

[17] Schulte L.H., May A. (2015). Headache research in 2014: Advancing migraine therapy. Lancet Neurol..

[18] Semenov I.A. (2015). Migraine headaches. Dis.-Month DM.

[19] Aoki K.R., Childers M.K. (2002). The Use of Botulinum Toxin Type a in Pain Management: A Clinecian’s Guide.

[20] Blumenfeld A., Silberstein S.D., Dodick D.W., Aurora S.K., Turkel C.C., Binder W.J. (2010). Method of injection of onabotulinumtoxina for chronic migraine: A safe, well-tolerated, and effective treatment paradigm based on the preempt clinical program. Headache.

[21] Hollanda L., Monteiro L., Melo A. (2014). Botulinum toxin type a for cephalic cutaneous allodynia in chronic migraine: A randomized, double-blinded, placebo-controlled trial. Neurol. Int..

[22] Poungvarin N. (2001). The first world report of botulinum A toxin injection for status migrainosus. J. Med. Assoc. Thail..

[23] Tamura B.M., Chang B. (2003). Botulinum toxin: Application into acupuncture points for migraine. Dermatol. Surg..

[24] Shao Y.F., Zhang Y., Zhao P., Yan W.J., Kong X.P., Fan L.L., Hou Y.P. (2013). Botulinum toxin type A therapy in migraine: Preclinical and clinical trials. Iran. Red Crescent Med. J..

[25] World Health Organization (2002). Acupuncture: Review and Analysis of Repotrs on Controlled Clinical Trials.

[26] Witt C.M., Reinhold T., Jena S., Brinkhaus B., Willich S.N. (2008). Cost-effectiveness of acupuncture treatment in patients with headache. Cephalalgia.

[27] Linde K., Allais G., Brinkhaus B., Manheimer E., Vickers A., White A.R. (2009). Acupuncture for migraine prophylaxis. Cochrane Database Syst. Rev..

[28] Karst M., Rollnik J.D., Fink M., Reinhard M., Piepenbrock S. (2000). Pressure pain threshold and needle acupuncture in chronic tension-type headache—A double-blind placebo-controlled study. Pain.

[29] Strudwick M.W., Hinks R.C., Choy S.T. (2007). Point injection as an alternative acupuncture technique—An exploratory study of responses in healthy subjects. Acupunct. Med..

[30] Wang S., Wang W., Li L. (1990). Treating neurotic headache by point-injection with novocain. J. Tradit. Chin. Med..

[31] Ga H., Choi J.H., Park C.H., Yoon H.J. (2007). Acupuncture needling *versus* lidocaine injection of trigger points in myofascial pain syndrome in elderly patients—A randomised trial. Acupunct. Med..

[32] Wong Y.K., Cheng J. (2003). A case series of temporomandibular disorders treated with acupuncture, occlusal splint and point injection therapy. Acupunct. Med..

[33] Yeom M.J., Lee H.C., Kim G.H., Shim I., Lee H.J., Hahm D.H. (2003). Therapeutic effects of hominis placenta injection into an acupuncture point on the inflammatory responses in subchondral bone region of adjuvant-induced polyarthritic rat. Biol. Pharm. Bull..

[34] Marx C., Silveira M.D., Selbach I., da Silva A.S., Braga L.M., Camassola M., Nardi N.B. (2014). Acupoint injection of autologous stromal vascular fraction and allogeneic adipose-derived stem cells to treat hip dysplasia in dogs. Stem Cells Int..

[35] Cady R., Schreiber C. (2008). Botulinum toxin type A as migraine preventive treatment in patients previously failing oral prophylactic treatment due to compliance issues. Headache.

[36] Barrientos N., Chana P. (2003). Botulinum toxin type A in prophylactic treatment of migraine headaches: A preliminary study. J. Headache Pain.

[37] Barbanti P., Egeo G., Fofi L., Aurilia C., Piroso S. (2015). Rationale for use of onabotulinum toxin A (BOTOX) in chronic migraine. Neurol. Sci..

[38] Silberstein S.D., Dodick D.W., Aurora S.K., Diener H.C., Degryse R.E., Lipton R.B., Turkel C.C. (2015). Percent of patients with chronic migraine who responded per onabotulinumtoxin A treatment cycle: PREEMPT. J. Neurol. Neurosurg. Psychiatr..

[39] Meng J., Wang J., Lawrence G., Dolly J.O. (2007). Synaptobrevin i mediates exocytosis of cgrp from sensory neurons and inhibition by botulinum toxins reflects their anti-nociceptive potential. J. Cell Sci..

[40] Burstein R., Zhang X., Levy D., Aoki K.R., Brin M.F. (2014). Selective inhibition of meningeal nociceptors by botulinum neurotoxin type A: Therapeutic implications for migraine and other pains. Cephalalgia.

[41] Du R., Wang Y., Liu X., Liu Z. (2015). Acupuncture for acute migraine attacks in adults: A systematic review protocol. BMJ Open.

[42] de Vernejoul P., Albarede P., Darras J.C. (1992). Nuclear medicine and acupuncture message transmission. J. Nuclear Med..

[43] Wang L., Cardini F., Zhao W., Regalia A.L., Wade C., Forcella E., Yu J. (2004). Vitamin k acupuncture pint injection for severe primary dysmenorrhea: An international pilot study. MedGenMed.

[44] Luo L., Chen W.J. (2001). Development of acu-injedtion treatment. J. Clin. Acupunct. Moxibust..

[45] Mathew N.T., Kailasam J., Meadors L. (2008). Predictors of response to botulinum toxin type A (BoNTA) in chronic daily headache. Headache.

[46] Gobel H. (2004). Botulinum toxin in migraine prophylaxis. J. Neurol..

[47] Jackson J.L., Kuriyama A., Hayashino Y. (2012). Botulinum toxin A for prophylactic treatment of migraine and tension headaches in adults: A meta-analysis. JAMA.

[48] Lipton R.B., Silberstein S.D. (2015). Episodic and chronic migraine headache: Breaking down barriers to optimal treatment and prevention. Headache.

[49] Headache Classification Subcommittee of the International Headache Society (2004). The international classification of headache disorders: 2nd edition. Cephalalgia.

[50] Sostak P., Krause P., Forderreuther S., Reinisch V., Straube A. (2007). Botulinum toxin type-A therapy in cluster headache: An open study. J. Headache Pain.

[51] Liu Y., Wang J. (2008). Illustration of Composed Acupoint in Acupuncture-Moxibustion Use.

[52] Shan X.F., Xu H., Cai Z.G., Wu L.L., Yu G.Y. (2013). Botulinum toxin A inhibits salivary secretion of rabbit submandibular gland. Int. J. Oral Sci..

